# Revision of the genus *Lechytia* Balzan, 1892 (Pseudoscorpiones, Chthoniidae) from China, with descriptions of two new species

**DOI:** 10.3897/BDJ.12.e122612

**Published:** 2024-04-19

**Authors:** Jianzhou Sun, Xiangbo Guo, Feng Zhang

**Affiliations:** 1 Key Laboratory of Zoological Systematics and Application, College of Life Sciences, Hebei University, Baoding, Hebei 071002, China Key Laboratory of Zoological Systematics and Application, College of Life Sciences, Hebei University Baoding, Hebei 071002 China; 2 Hebei Basic Science Center for Biotic Interaction, Hebei University, Baoding, Hebei 071002, China Hebei Basic Science Center for Biotic Interaction, Hebei University Baoding, Hebei 071002 China

**Keywords:** new species, Lechytiinae, taxonomy, Xizang

## Abstract

**Background:**

The *hoffi* species-group previously comprised only two species, *Lechytiahoffi* Muchmore, 1975 and *Lechytiayulongensis* Zhang and Zhang, 2014, of which *L.yulongensis* is distributed in China.

**New information:**

Three species of the genus *Lechytia* are described from China: *Lechytiaacutidentata* **sp. nov.** and *Lechytiadepressidentata* **sp. nov.** from Xizang Autonomous Region and *L.yulongensis* from Yunnan Province. The female of *L.yulongensis* is reported for the first time. In addition, a key to the *hoffi* species-group is provided.

## Introduction

The pseudoscorpion genus *Lechytia* Balzan, 1892 was established for a Neotropic species, *Lechytiachthoniiformis* (Balzan 1887), which was originally placed in the genus *Roncus* L. Koch, 1873 (Balzan 1892). From then on, *Lechytia* belonged to Chthoniidae Daday, 1889, until Harvey (1992) elevated it to the family level due to the lacking of an elliptical areole on the rallum and the short inter-maxillary jugum. Subsequently, it was reduced to a subfamily (Lechytiinae) within Chthoniidae, based on phylogenetic analyses using transcriptome data (Benavides et al. 2019). The most peculiar diagnostic feature of Lechytiinae is the trichobothria *eb* and *esb* situated on the dorsum of the chelal hand, while these trichobothria are situated at the base of the fixed chelal finger in all other members of Chthoniidae (Harvey 2006, Christophoryová and Krajčovičová 2020).

Currently, there are 26 reported *Lechytia* species widespread in most parts of the world, with six species in Africa, seven in Asia (including the Pacific), 11 in the Americas (including one fossilised species) and two in Oceania (WPC 2024). *Lechytia* contains two species-groups: *arborea* species-group for six species from the Americas, Oceania and the Pacific and *hoffi* species-group for two species from North America and Asia (Muchmore 1975, Muchmore 2000, Harvey 2006, Zhang and Zhang 2014, Christophoryová and Krajčovičová 2020). The remaining 18 species are not placed in the two known species-groups. The *hoffi* (*arborea*) species-group is diagnosed as follows: well-developed (strongly reduced) chelal teeth, simple (bifurcate) distal seta on pedipalpal coxa, tergite XI with chaetotaxy T2T (1T2T1) and male galea nearly as well developed as in female (male galea reduced).

At present, only one *Lechytia* species, *L.yulongensis* Zhang and Zhang, 2014, has been reported from China (Zhang and Zhang 2014), which belongs in the *hoffi* species-group together with *L.hoffi*. Herein, two new *Lechytia* species from China are described. In addition, the female of *L.yulongensis* is reported for the first time.

## Materials and methods

**Specimen preparation and examination.** The specimens examined for this study are preserved in 75% ethyl alcohol and in a refrigerator at -20°C and deposited in the Museum of Hebei University (MHBU) (Baoding, China). Photographs, drawings and measurements were taken using a Leica M205A stereo-microscope equipped with a Leica DFC550 camera and the Inkscape software (Ver. 1.0.2.0). Detailed examination was carried out with an Olympus BX53 general optical microscope. All images were edited and formatted using Adobe Photoshop 2017.

**Preparation for scanning electron microscopy (SEM).** The samples were placed in 95% ethanol for one hour, followed by placement in 100% ethanol and finally switched to fresh 100% ethanol overnight. Each sample was proceeded to critical point drying (POLARON E3000), using Carbon Dioxide (CO_2_) as transitional fluid. The specimen was processed as above and then the sample was photographed using a SEM.

**Terminology.** Terminology and measurements follow Chamberlin (1931) with some small modifications to the terminology of trichobothria (Harvey 1992, Judson 2007) and chelicera (Judson 2007). The chela and legs are measured in lateral view and others are taken in dorsal view. All measurements are given in mm unless noted otherwise. Proportions and measurements of chelicerae, carapace and pedipalps correspond to length/breadth and those of legs to length/depth.

The following abbreviations are used in the text: for the chelal trichobothria: ***b*** = basal; ***sb*** = sub-basal; ***st*** = subterminal; ***t*** = terminal; ***ib*** = interior basal; ***isb*** = interior sub-basal; ***ist*** = interior sub-terminal; ***it*** = interior terminal; ***eb*** = exterior basal; ***esb*** = exterior sub-basal; ***est*** = exterior sub-terminal; ***et*** = exterior terminal. For additional abbreviations: *dx*, duplex trichobothria.

## Taxon treatments

### Lechytia acutidentata sp. nov.

CDF320A8-04B4-589F-87D4-E933A44F33D9

34A4EC3B-9F38-47B3-96E7-6289B5C700BA

#### Materials

- **Type status:** Holotype. **Occurrence:** recordedBy: Xiangbo Guo, Bo Liu & Haibin Zhang; sex: female; lifeStage: adult; occurrenceID: 3E280ECF-CA68-54EE-A007-D55C009D6828; **Taxon:** scientificName: *Lechytiaacutidentata*; **Location:** country: China; stateProvince: Xizang Autonomous Region; county: Jilong; locality: Jilong Town, Rema Village, under bark; verbatimElevation: 3272 m; verbatimCoordinates: 28.455680°N, 85.196614°E; **Identification:** identifiedBy: Jianzhou Sun; **Event:** eventID: HBUARA#2023-880; year: 2023; month: August; day: 2; **Record Level:** institutionID: the Museum of Hebei University (MHBU); institutionCode: Ps.-MHBU-XZ2023080201
- **Type status:** Paratype. **Occurrence:** recordedBy: Xiangbo Guo, Bo Liu & Haibin Zhang; sex: 1 male, 31 females; lifeStage: adult; occurrenceID: 2053BC16-16DC-5FC4-A9C5-76DA95B44FEC; **Taxon:** scientificName: *Lechytiaacutidentata*; **Location:** country: China; stateProvince: Xizang Autonomous Region; county: Jilong; locality: Jilong Town, Rema Village, under bark; verbatimElevation: 3272 m; verbatimCoordinates: 28.455680°N, 85.196614°E; **Identification:** identifiedBy: Jianzhou Sun; **Event:** eventID: HBUARA#2023-880; year: 2023; month: August; day: 2; **Record Level:** institutionID: the Museum of Hebei University; institutionCode: male (Ps.-MHBU-XZ2023080202) and 31 females (Ps.-MHBU-XZ2023080203–033)

#### Description

**Figure 1.**
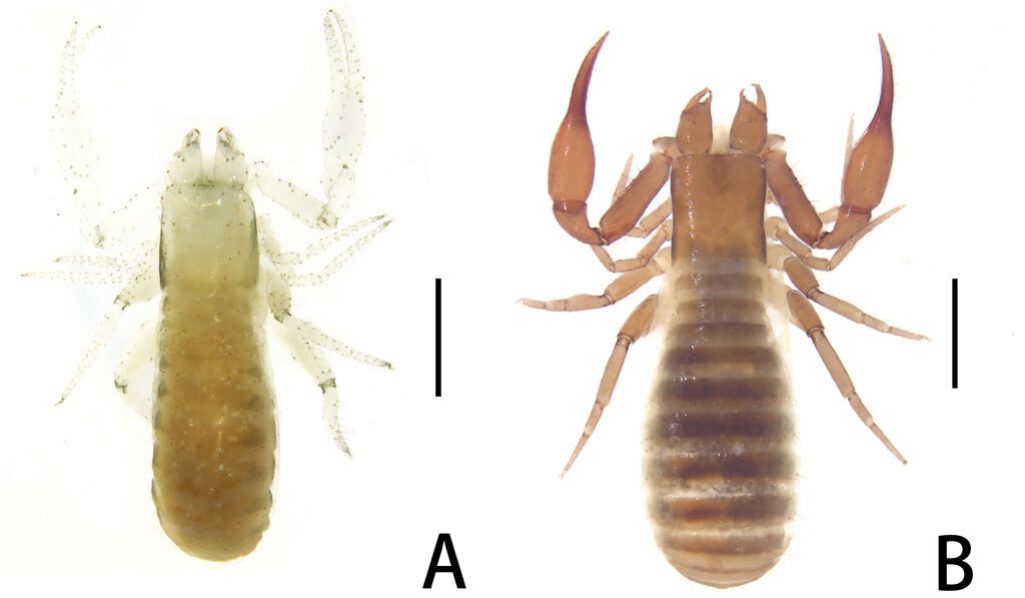
*Lechytiaacutidentata* sp. nov. **A** Paratype male (dorsal view); **B** Holotype female (dorsal view). Scale bars: 0.50 mm.

**Figure 2.**
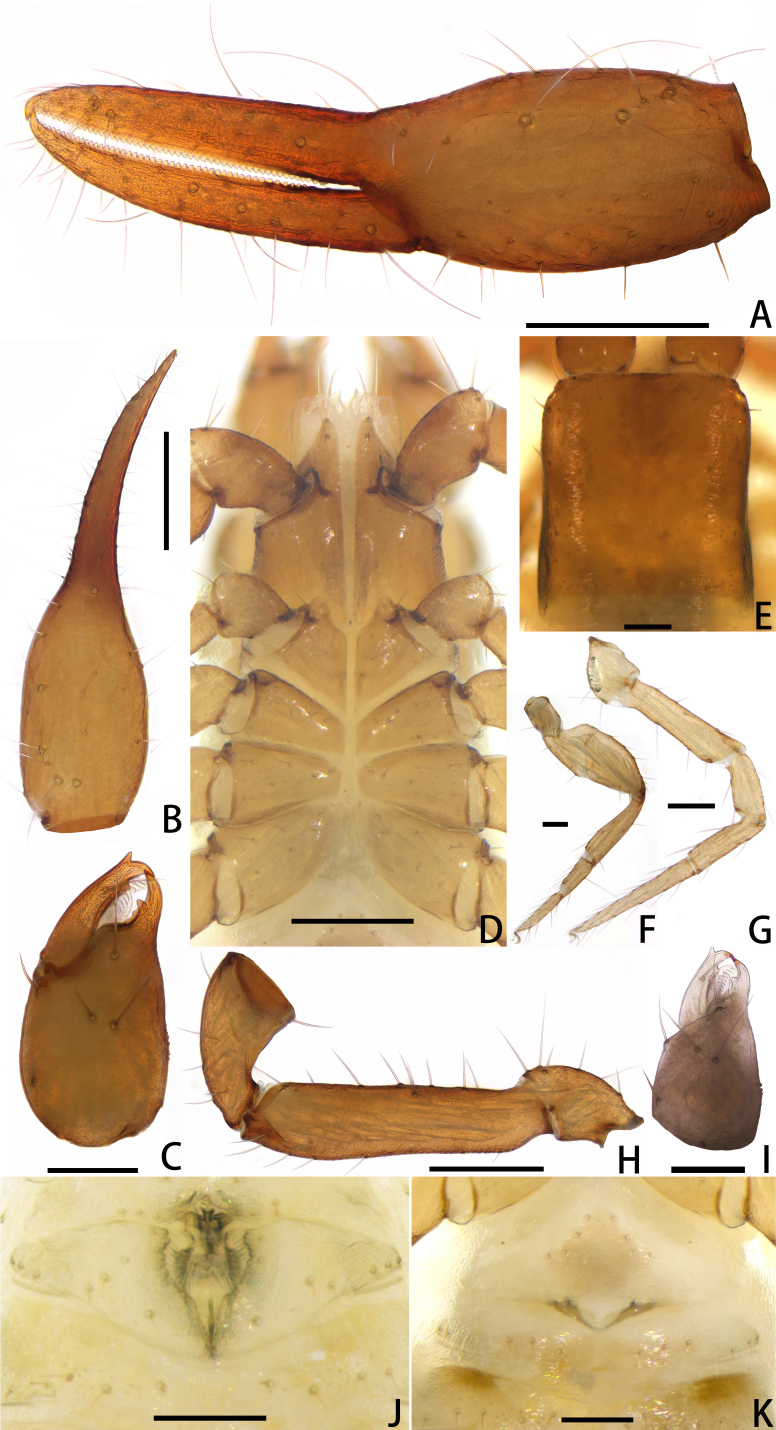
*Lechytiaacutidentata* sp. nov., holotype female (A–H, K), paratype male (I, J): **A** Female left chela (lateral view); **B** Left chela (dorsal view); **C** Left chelicera (dorsal view); **D** Coxal area and manducatory process (ventral view); **E** Carapace (dorsal view); **F** Leg Ⅳ (lateral view); **G** Leg Ⅰ (lateral view); **H** Left pedipalp (minus chela, dorsal view); **I** Male left chelicera (dorsal view); **J** Male genital area (ventral view); **K** Female genital area (ventral view). Scale bars: 0.20 mm (A, B, D, H); 0.10 mm (C, E–G, I–K).

**Figure 3.**
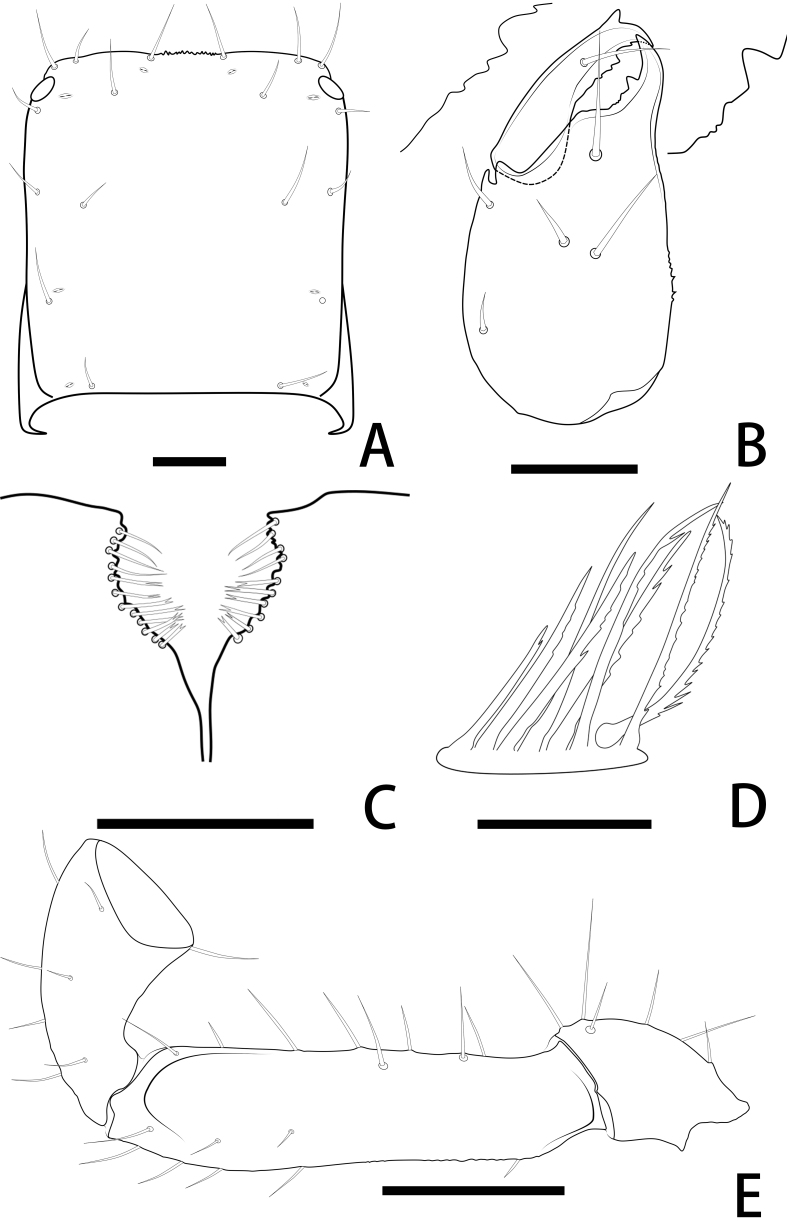
*Lechytiaacutidentata* sp. nov., holotype female: **A** Carapace (dorsal view); **B** Left chelicera (dorsal view), with details of teeth; **C** Chaetotaxy of genital area (sternites II–III) (male); **D** Rallum; **E** Left pedipalp (minus chela, dorsal view). Scale bars: 0.10 mm (A–C); 0.05 mm (D); 0.20 mm (E).

**Figure 4.**
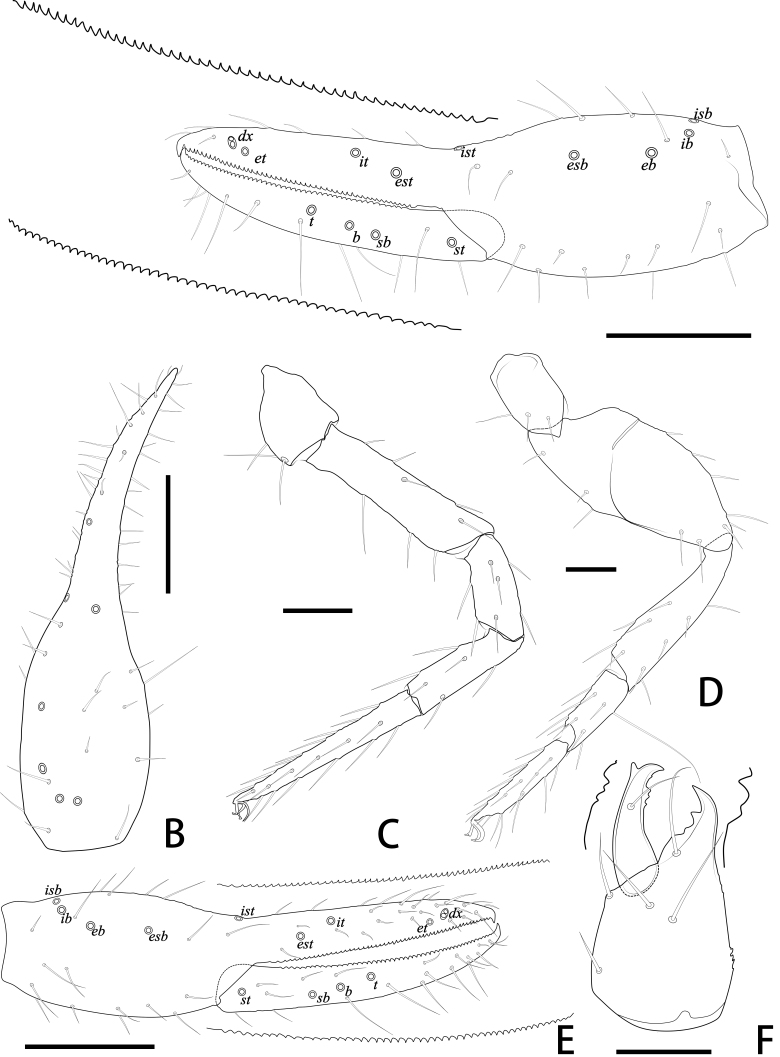
*Lechytiaacutidentata* sp. nov., holotype female (A–D), paratype male (E, F): **A** Left chela (lateral view), with details of teeth and trichobothrial pattern; **B** Left chela (dorsal view); **C** Leg I (lateral view); **D** Leg IV (lateral view); **E** Left chela (lateral view), with details of teeth and trichobothrial pattern; **F** Left chelicera (dorsal view), with details of teeth. Scale bars: 0.20 mm (A, B, E); 0.10 mm (C, D, F).

Females (holotype and paratypes) (Fig. 1B, Fig. 2A–H, K and Figs 3, 4A–D).

**Figure 5.**
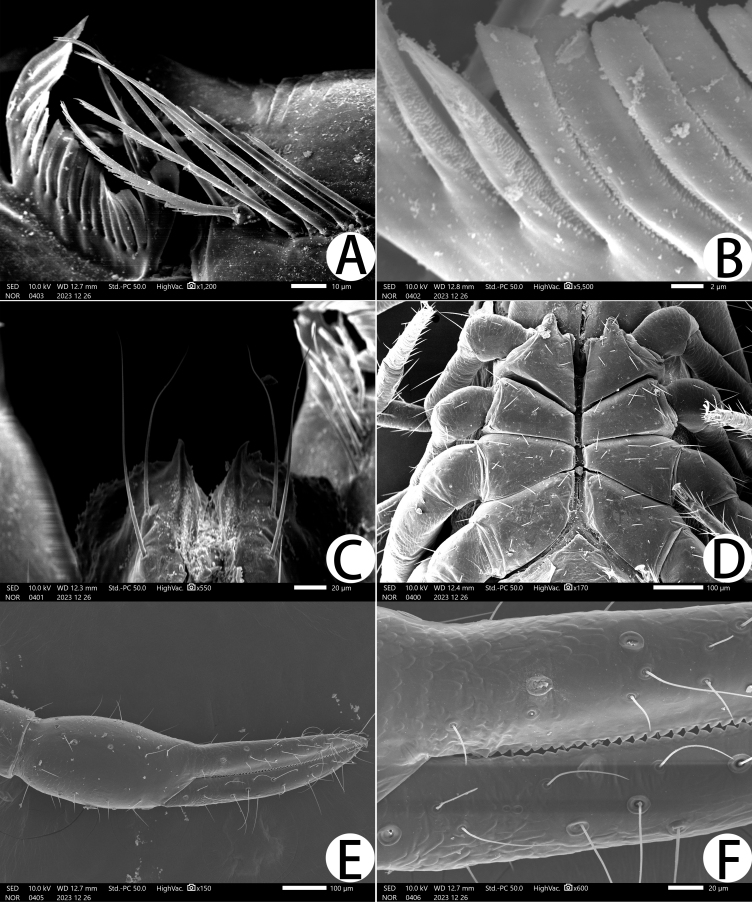
*Lechytiaacutidentata* sp. nov., paratype female: **A** Rallum; **B** Serrula exterior; **C** Manducatory process (ventral view); **D** Coxal area (ventral view); **E** Right chela (lateral view), with details of teeth and trichobothrial pattern; **F** Chelal basal details of teeth and trichobothrial.

**Cephalothorax** (Fig. 2D, E and Fig. 3A): carapace nearly subquadrate, 0.98–1.09 times longer than broad; anterior margin denticulate; without epistome; two small corneate eyes; with 18 setae arranged 6: 4: 4: 2: 2, most setae heavy, long and gently curved; with four pairs of lyrifissures, first pair situated antero-medially, the second pair situated interno-underneath to the eyes, the third pair situated slightly interior to the sole pair of setae of the intermediate row and the fourth pair situated exterior to the sole pair of setae of the posterior row. Manducatory process with two acuminate distal setae, about equal in length, the distal setae terminally acuminate (Fig. 5C); apex of coxa I with a triangular apical projection (Fig. 5D); coxal spines and intercoxal tubercle absent. Chaetotaxy of coxae: P 5, Ⅰ 7, II 7, III 7, IV 7.

**Chelicera** (Fig. 2C and Fig. 3B): 1.39–1.56 times longer than broad; five setae present on hand, all setae acuminate, ventrobasal setae shorter than others; movable finger with one medial seta. Cheliceral palm has moderate wrinkles on both ventral and dorsal sides. Fixed finger with one large tooth and two roughened ridges proximally; movable finger with an acute apical tooth and four pointed, conspicuous middle teeth; galea shaped like a tooth (♂♀) (Fig. 2C, I, Fig. 3B and Fig. 4F). Serrula exterior with 17–18 blades, smooth surface and side creases (Fig. 5B). Rallum with eight blades, subdistal blade strongly recumbent, others in straight row (Fig. 3D and Fig. 5A).

**Pedipalp** (Fig. 2A, B, H, Fig. 3E, Fig. 4A and B): trochanter 1.54–1.91, femur 3.86–4.73, patella 1.76–1.88, chela 3.59–3.90, hand 1.54–1.75 times longer than broad; femur 1.73–1.80 times longer than patella; movable chelal finger 1.28–1.42 times longer than hand and 0.57–0.61 times longer than chela. Setae generally long and acuminate. Fixed chelal finger and hand with eight trichobothria, *ib*, *isb*, *eb* and *esb* on dorsum of hand, *ib* and *isb* basally, *esb* submedially, *eb* closer to *ib* and *isb* than to *esb*; *ist* situated basally on fixed finger, *est* and *it* situated submedially and medially on fixed finger, *et* and *dx* distally; movable chelal finger with four trichobothria, *b* closer to *sb* than to *t*, *sb* closer *b* than to *st*; *b* and *sb* situated more than one diameter apart (Fig. 2A, Fig. 4A, Fig. 5E and F); sensilla absent. Both chelal fingers with a row of teeth: fixed finger with 50–52 developed retrorse, pointed teeth; movable finger with 48–53 upright, pointed teeth.

**Opisthosoma**: tergites and sternites undivided; setae acuminate. Tergal chaetotaxy I–Ⅻ: 6: 4–6: 5–6: 6–7: 6: 6: 6: 6: 6: 4–6: T2T: 0. Sternal chaetotaxy Ⅳ–Ⅻ: 14–16: 12: 10: 10–12: 8–10: 8: 5–6: -: 2. Genital area weakly sclerotised with U-shaped frame (Fig. 2K).

**Legs** (Fig. 2F, G, Fig. 4C and D): leg Ⅰ: femur 1.56–1.93 times longer than patella; tarsus 1.61–1.76 times longer than tibia. Leg Ⅳ: femoropatella 2.10–2.50 times longer than deep; tibia 3.78–4.25 times longer than deep; with sub-basal tactile setae on basitarsal segments. Arolium slightly shorter than the claws, not divided; claws simple.

**Adult male** (paratype) (Fig. 1A, Fig. 2J, Fig. 3C, Fig. 4E and F). Mostly same as females, but a little smaller on average; tergal chaetotaxy Ⅰ–Ⅻ: 6: 6: 6: 6: 6: 6: 6: 6: 6: 4: T2T: 0; sternal chaetotaxy Ⅳ–Ⅻ: 14: 10: 8: 8: 8: 8: 6: -: 2. Genital region: partial setaes bifurcate.

**Dimensions** (length/breadth or, in the case of the legs, length/depth in mm; ratios in parentheses). Male: body length 1.71. Pedipalps: trochanter 0.21/0.11 (1.91), femur 0.49/0.12 (4.08), patella 0.29/0.17 (1.71), chela 0.75/0.19 (3.95), hand 0.32/0.19 (1.68), movable chelal finger length 0.44. Chelicera 0.24/0.16 (1.50), movable finger length 0.12. Carapace 0.45/0.39 (1.15). Leg I: trochanter 0.18/0.11 (1.64), femur 0.29/0.06 (4.83), patella 0.13/0.05 (2.60), tibia 0.12/0.05 (2.40), tarsus 0.25/0.04 (6.25). Leg Ⅳ: trochanter 0.16/0.09 (1.78), femoropatella 0.37/0.18 (2.06), tibia 0.33/0.09 (3.67), basitarsus 0.16/0.06 (2.67), telotarsus 0.22/0.04 (5.50).

Females: body length 1.79–2.07. Pedipalps: trochanter 0.20–0.21/0.11–0.13 (1.54–1.91), femur 0.51–0.54/0.11–0.14 (3.86–4.73), patella 0.29–0.30/0.16–0.17(1.76–1.88), chela 0.77–0.82/0.20–0.22 (3.59–3.90), hand 0.32–0.37/0.20–0.22 (1.54–1.75), movable chelal finger length 0.45–0.48. Chelicera 0.25–0.29/0.18–0.20 (1.39–1.56), movable finger length 0.15–0.16. Carapace 0.43–0.47/0.42–0.46 (0.98–1.09). Leg I: trochanter 0.11–0.14/0.08–0.11 (1.18–1.75), femur 0.25–0.29/0.06–0.07 (3.71–4.83), patella 0.15–0.16/0.05–0.07 (2.14–3.00), tibia 0.12–0.17/0.05 (3.20–3.60), tarsus 0.27–0.30/0.04–0.05 (5.60–7.50). Leg Ⅳ: trochanter 0.15–0.19/0.09–0.14 (1.25–1.78), femoropatella 0.42–0.45/0.18–0.20 (2.10–2.50), tibia 0.33–0.35/0.08–0.09 (3.78–4.25), basitarsus 0.16–0.18/0.06–0.07 (2.29–2.83), telotarsus 0.23–0.24/0.04–0.05 (4.60–6.00).

#### Diagnosis

The new species belongs to the *hoffi* species-group and is characterised by the following combination of characteristics: trichobothria *b* and *sb* situated more than one areolar diameter apart; movable chelal finger with upright and point teeth; palpal chela 3.59–3.90 (♀), 3.95 (♂) and palpal hand 1.54–1.75 (♀), 1.68 (♂) longer than broad; palpal femur 0.51–0.54 mm (♀), 0.49 mm (♂), palpal hand 0.33–0.37 mm (♀), 0.32 mm (♂) and chelal movable finger 0.45–0.48 mm (♀), 0.44 mm (♂) long.

*Lechytiaacutidentata* **sp. nov.** most resembles *L.yulongensis* due to trichobothria *b* and *sb* on movable chelal finger situated more than one areolar diameter apart. However, the new species differs from *L.yulongensis* in the pattern of teeth of fixed chelal finger with upright and point teeth in *L.acutidentata* **sp. nov.**, but retrorse and point teeth in *L.yulongensis* (*Zhang and Zhang 2014*).

#### Etymology

The specific name is derived from a combination of the Latin words “*acutus*” and “*dentatus*”, meaning pointed and toothed, respectively, which refers to the pointed teeth on the movable chelal fingers.

#### Distribution

China (Xizang Autonomous Region).

#### Ecology

**Figure 6.**
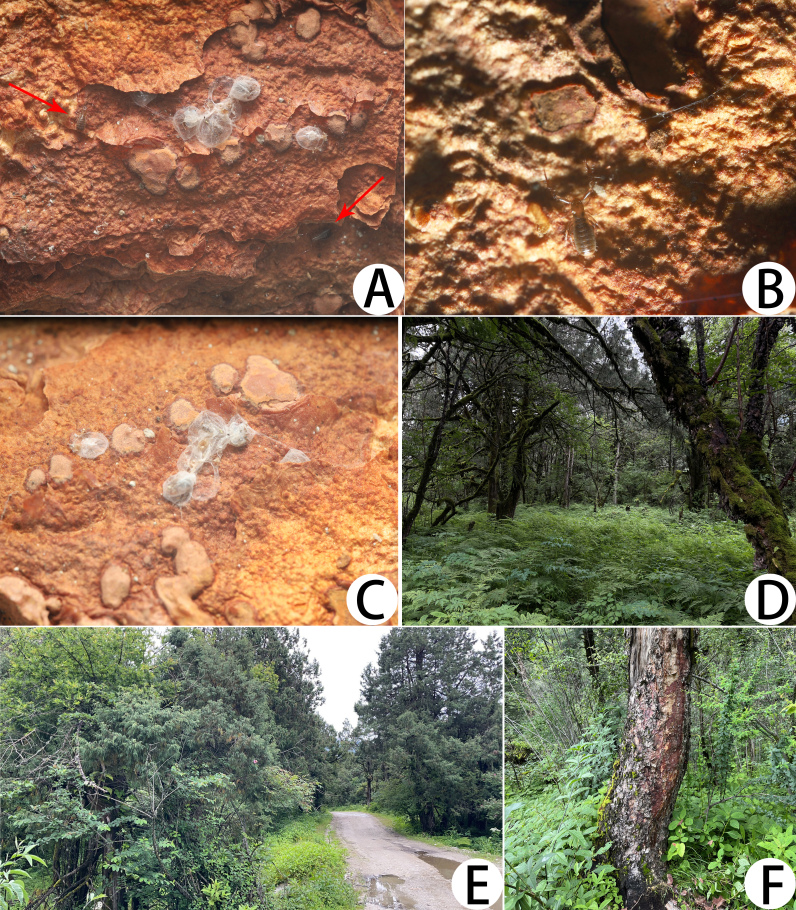
Type locality of *Lechytiaacutidentata* sp. nov. **A** Numerous *L.acutidentata* sp. nov. and silk nests; **B** A living of *L.acutidentata* sp. nov. in its natural environment; **C** Silk nests of *L.acutidentata* sp. nov.; **D–F**. The habitat where *L.acutidentata* sp. nov. specimens were collected.

All specimens were collected under bark in denser forests (Fig. 6).

#### Biology

Their rounded nests are built by thin silks and had a thicker centre (Fig. 6A and C). Some silk nests are close together, but independent from each other (Fig. 6A).

#### Notes

Generally, the holotypes of *Lechytia* species are male specimens. Here, we chose a female specimen as the holotype of *L.acutidentata*, because the sole male specimen has a rather transparent and brittle body. It was probably caught and soaked in alcohol straight after just moulting.

### Lechytia depressidentata sp. nov.

B8F8E5CF-C93D-5FE9-B45A-E1299107D022

8ED81D43-CDF7-4AD1-ACF3-1B7C0DB6E560

#### Materials

- **Type status:** Holotype. **Occurrence:** recordedBy: Xiangbo Guo, Bo Liu & Haibin Zhang; sex: male; lifeStage: adult; occurrenceID: D3736A6C-02E2-54FE-9B99-12FF869FC5AE; **Taxon:** scientificName: *Lechytiadepressidentata*; **Location:** country: China; stateProvince: Xizang Autonomous Region; county: Jilong; locality: Jilong Town, Kongsang Bridge, under bark; verbatimElevation: 2697 m; verbatimCoordinates: 28.384151°N, 85.353469°E; **Identification:** identifiedBy: Jianzhou Sun; **Event:** eventID: HBUARA#2023-885; year: 2023; month: August; day: 4; **Record Level:** institutionID: the Museum of Hebei University; institutionCode: Ps.-MHBU-XZ2023080401
- **Type status:** Paratype. **Occurrence:** recordedBy: Xiangbo Guo, Bo Liu & Haibin Zhang; sex: 2males, 6 females; lifeStage: adult; occurrenceID: 634D1277-955C-5E1E-A3C3-ABE60C6365A3; **Taxon:** scientificName: *Lechytiadepressidentata*; **Location:** country: China; stateProvince: Xizang Autonomous Region; county: Jilong; locality: Jilong Town, Kongsang Bridge, under bark; verbatimElevation: 2697 m; verbatimCoordinates: 28.384151°N, 85.353469°E; **Identification:** identifiedBy: Jianzhou Sun; **Event:** eventID: HBUARA#2023-885; year: 2023; month: August; day: 4; **Record Level:** institutionID: the Museum of Hebei University; institutionCode: 2 males (Ps.-MHBU-XZ2023080402–03) and 6 females (Ps.-MHBU-XZ2023080404–09)

#### Description

**Figure 7.**
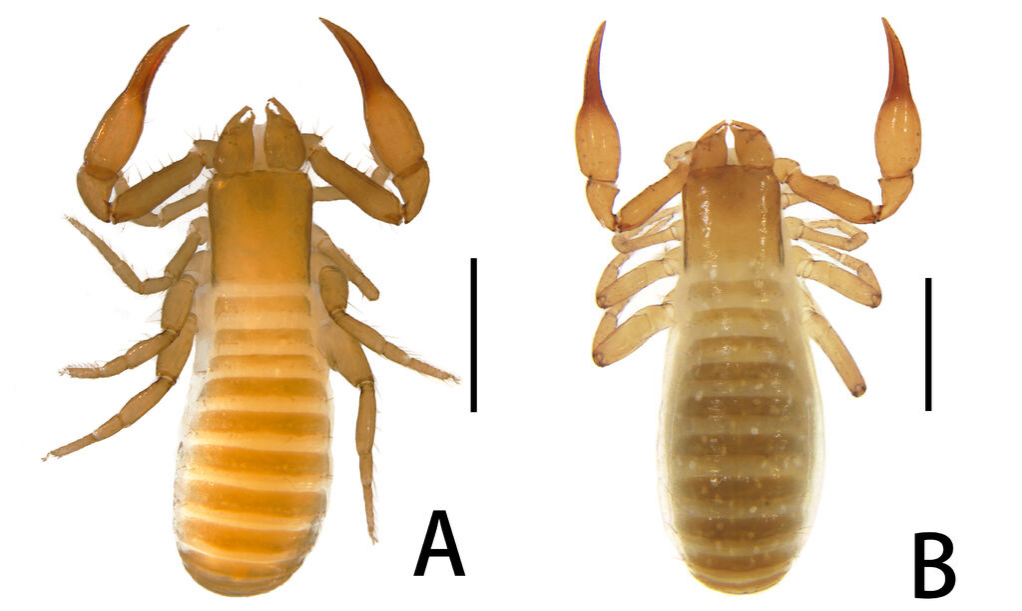
*Lechytiadepressidentata* sp. nov. **A**. Holotype male (dorsal view); **B** Paratype female (dorsal view). Scale bars: 0.50 mm.

**Figure 8.**
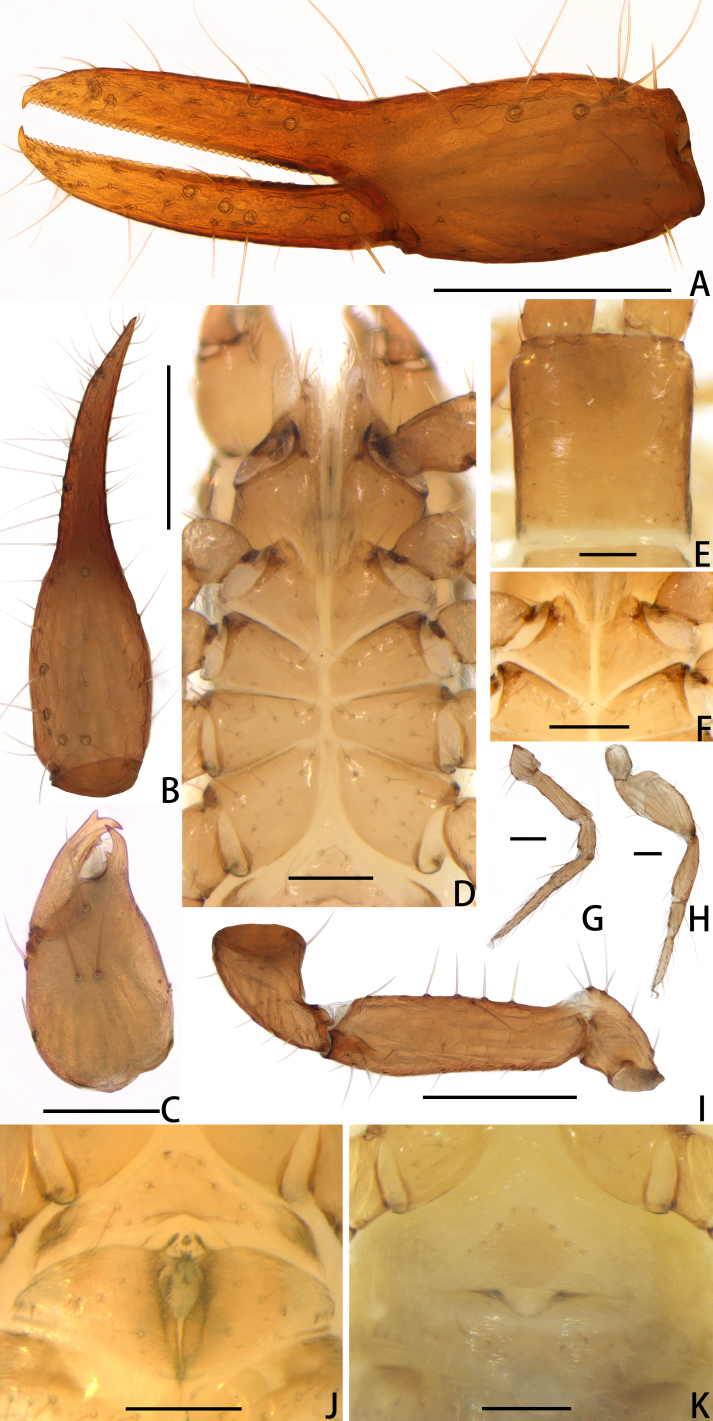
*Lechytiadepressidentata* sp. nov., holotype male (A–J), paratype female (K): **A** Left chela (lateral view); **B** Left chela (dorsal view); **C** Left chelicera (dorsal view); **D** Coxal area and manducatory process (ventral view); **E** Carapace (dorsal view); **F** Apex of coxa I; **G** Leg Ⅰ (lateral view); **H** Leg Ⅳ (lateral view); **I** Left pedipalp (minus chela, dorsal view); **J** Male genital area (ventral view); **K** Female genital area (ventral view). Scale bars: 0.20 mm (A, B, I); 0.10 mm (C–H, J, K).

**Figure 9.**
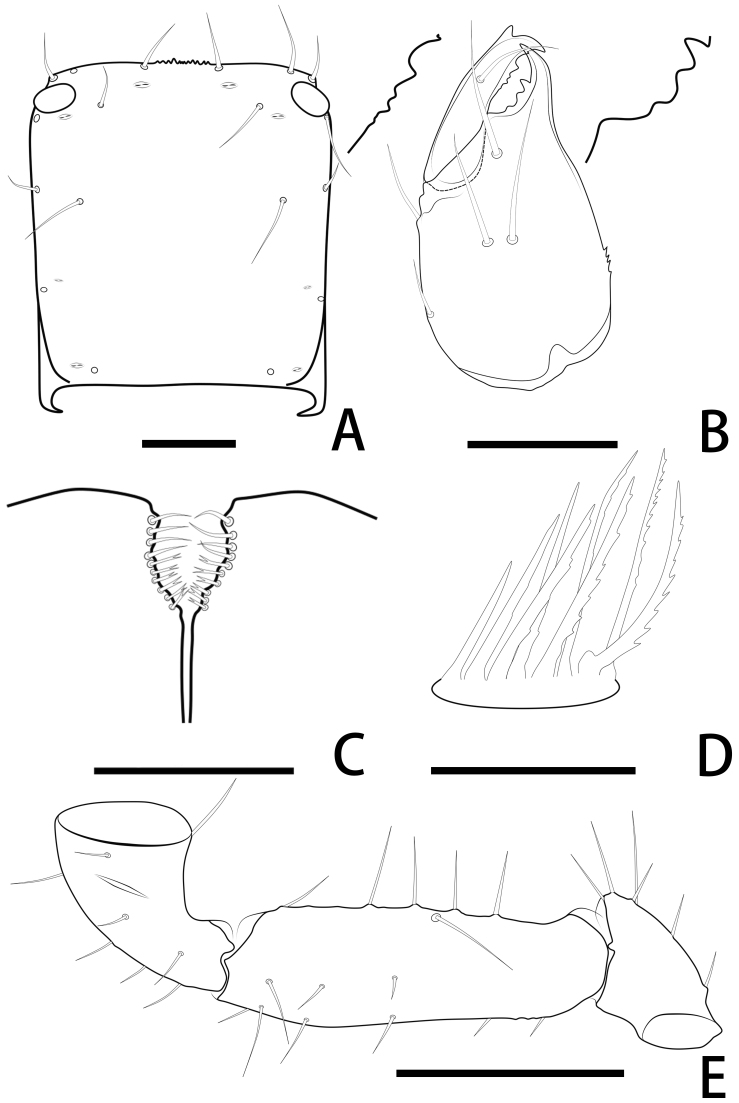
*Lechytiadepressidentata* sp. nov., holotype male: **A** Carapace (dorsal view); **B** Left chelicera (dorsal view), with details of teeth; **C** Chaetotaxy of genital area (sternites II–III) (male); **D** Rallum; **E** Left pedipalp (minus chela, dorsal view). Scale bars: 0.10 mm (A–C, I); 0.05 mm (D); 0.20 mm (E).

**Figure 10.**
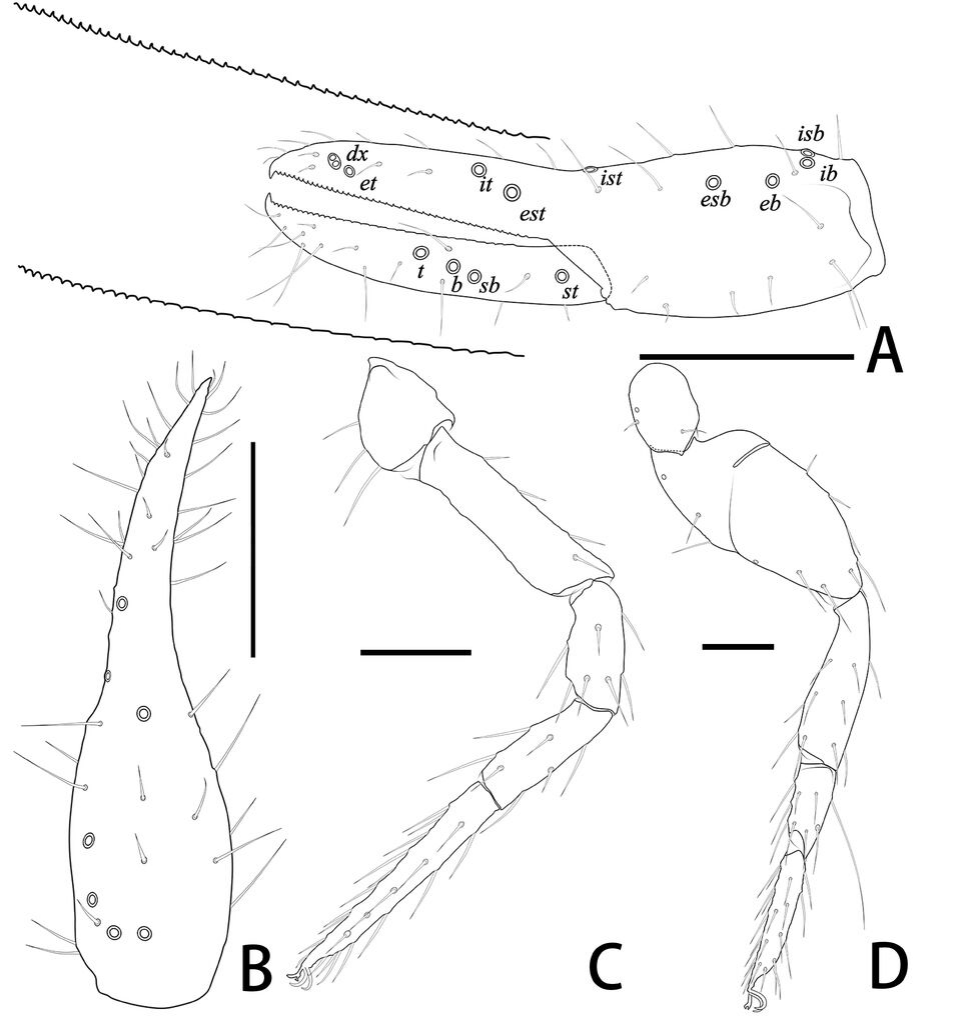
*Lechytiadepressidentata* sp. nov., holotype female (A–D), paratype male (E, F): **A** Left chela (lateral view), with details of teeth and trichobothrial pattern; **B** Left chela (dorsal view); **C** Leg I (lateral view); **D** Leg IV (lateral view). Scale bars: 0.20 mm (A, B); 0.10 mm (C, D).

Males (holotype and paratypes) (Fig. 7A, Fig. 8A–J and Figs 9, 10).

**Figure 11.**
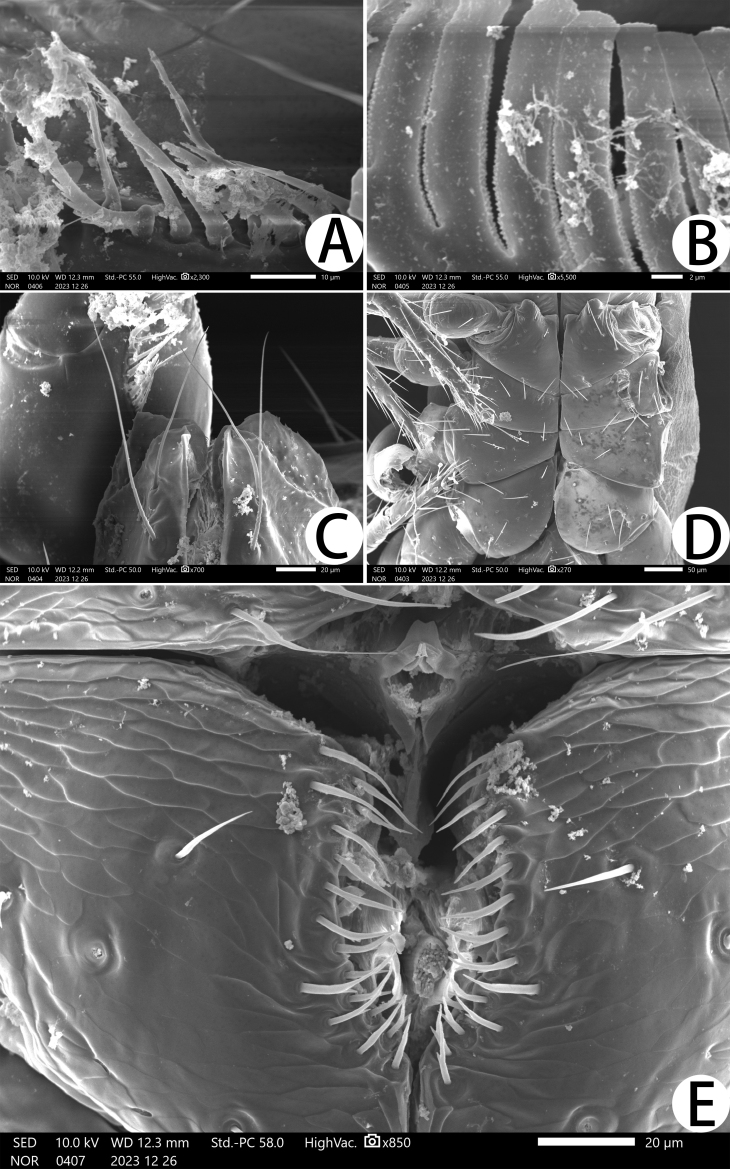
*Lechytiadepressidentata* **sp. nov.**, paratype male: **A** Rallum; **B** Serrula exterior; **C** Manducatory process (ventral view); **D** Coxal area (ventral view); **E** Male genital area (ventral view).

**Cephalothorax** (Fig. 8D–F and Fig. 9A): carapace nearly subquadrate, 0.91–1.09 times longer than broad; anterior margin denticulate; without epistome; two small corneate eyes; with 18 setae arranged 6: 4: 4: 2: 2, most setae heavy, long and gently curved; with four pairs of lyrifissures, first pair situated antero-medially, the second pair situated interno-underneath to the eyes, the third pair situated slightly interior to the sole pair of setae of the intermediate row and the fourth pair situated exterior to the sole pair of setae of the posterior row. Manducatory process with two acuminate distal setae, about equal in length, the distal setae terminally acuminate (Fig. 11C); apex of coxa I with a triangular apical projection; coxal spines and intercoxal tubercle absent. Chaetotaxy of coxae: P 5, Ⅰ 7, II 7, III 7, IV 7.

**Chelicera** (Fig. 8C and Fig. 9B): 1.40–1.53 times longer than broad; five setae present on hand, all setae acuminate, ventrobasal setae shorter than others; movable finger with one medial seta. Cheliceral palm has moderate wrinkles on both ventral and dorsal sides. Fixed finger with one large tooth and two or three roughened ridges proximally; movable finger with an acute apical tooth and three or four pointed, conspicuous middle teeth; galea shaped like a tooth (Fig. 9B). Serrula exterior with 17–18 blades, smooth surface and side creases (Fig. 11B). Rallum with eight blades, subdistal blade strongly recumbent, others in straight row (Fig. 9D).

**Pedipalp** (Fig. 8A–B, I, Fig. 9E, Fig. 10A and B): trochanter 1.20–1.78, femur 3.45–3.60, patella 1.75–1.83, chela 3.56–3.73, hand 1.56–1.67 times longer than broad; femur 1.64–1.81 times longer than patella; movable chelal finger 1.36–1.38 times longer than hand and 0.59–0.63 times longer than chela. Setae generally long and acuminate. Fixed chelal finger and hand with eight trichobothria, *ib*, *isb*, *eb* and *esb* on dorsum of hand, *ib* and *isb* basally, *esb* submedially, *eb* closer to *ib* and *isb* than to *esb*; *ist* situated basally on fixed finger, *est* and *it* situated submedially and medially on fixed finger, *et* and *dx* distally; movable chelal finger with four trichobothria, *b* closer to *sb* than to *t*, *sb* closer *b* than to *st*; *b* and *sb* situated less than one areolar diameter apart (Fig. 8A and Fig. 10A); sensilla absent. Both chelal fingers with a row of teeth: fixed finger with 42–44 developed retrorse, pointed teeth; movable finger with 13–14 small, pointed teeth at distal end, followed proximally by 18–22 flattened teeth.

**Opisthosoma**: tergites and sternites undivided; setae acuminate. Tergal chaetotaxy I–Ⅻ: 6: 6: 6: 6: 6: 6: 6: 6: 6: 4: T2T: 0. Sternal chaetotaxy Ⅱ–Ⅻ: 10: 14–16: 11–14: 10–12: 10: 8–10: 6–8: 6: -: 2. Genital region: partial setaes bifurcate (Fig. 11F).

**Legs** (Fig. 8G, H, Fig. 10C and D): leg Ⅰ: femur 1.67–1.82 times longer than patella; tarsus 1.92 times longer than tibia. Leg Ⅳ: femoropatella 2.19–2.40 times longer than deep; tibia 3.71–4.17 times longer than deep; with subbasal tactile setae on basitarsal segments. Arolium slightly shorter than the claws, not divided; claws simple.

**Adult female** (paratypes) (Fig. 7B and Fig. 8K). Mostly same as males, but a little larger on average; tergal chaetotaxy Ⅰ–Ⅻ: 6: 6: 6: 6: 6: 6: 6: 6: 6: 4: T2T: 0; sternal chaetotaxy Ⅱ–Ⅻ: 8: 12: 14: 12: 10: 10: 8: 6–8: 6: -: 2. Genital area weakly sclerotised with U-shaped frame.

**Dimensions** (length/breadth or, in the case of the legs, length/depth in mm; ratios in parentheses). Males: body length 1.36–1.41. Pedipalps: trochanter 0.12–0.16/0.09–0.10 (1.40–1.53), femur 0.36–0.38/0.10–0.11 (3.45–3.60), patella 0.21–0.22/0.12 (1.75–1.83), chela 0.56–0.57/0.15–0.16 (3.56–3.73), hand 0.24–0.25/0.15–0.16 (1.56–1.67), movable chelal finger length 0.33–0.35. Chelicera 0.21–0.23/0.15 (1.40–1.53), movable finger length 0.12. Carapace 0.32–0.35/0.32–0.35 (0.91–1.09). Leg I: trochanter 0.09–0.10/0.06–0.08 (1.13–1.50), femur 0.20/0.05 (4.00), patella 0.11–0.12/0.04–0.05 (2.20–2.75), tibia 0.12/0.04 (3.00), tarsus 0.23/0.03 (7.67). Leg Ⅳ: trochanter 0.12–0.14/0.10 (1.20–1.40), femoropatella 0.35–0.36/0.15–0.16 (2.19–2.40), tibia 0.23–0.26/0.06–0.07 (3.71–4.17), basitarsus 0.12–0.13/0.05 (2.40–2.60), telotarsus 0.19–0.20/0.03–0.04 (4.75–6.67).

Females: body length 1.50–1.57. Pedipalps: trochanter 0.16/0.09 (1.78), femur 0.38–0.39/0.12 (3.17–3.25), patella 0.22–0.23/0.13 (1.69–1.77), chela 0.59–0.60/0.17–0.18 (3.33–3.47), hand 0.26–0.27/0.17–0.18 (1.50–1.53), movable chelal finger length 0.35–0.36. Chelicera 0.22/0.16–0.17 (1.29–1.38), movable finger length 0.13. Carapace 0.38–0.40/0.37–0.38 (1.03–1.05). Leg I: trochanter 0.09/0.07–0.09 (1.00–1.29), femur 0.19–0.22/0.05–0.06 (3.67–3.80), patella 0.11–0.12/0.06 (1.83–2.00), tibia 0.12–0.13/0.04 (3.00–3.25), tarsus 0.23–0.24/0.03 (7.67–8.00). Leg Ⅳ: trochanter 0.14–0.15/0.09–0.12 (1.25–1.56), femoropatella 0.36–0.37/0.15–0.16 (2.31–2.40), tibia 0.26–0.28/0.08 (3.25–3.50), basitarsus 0.13/0.06 (2.17), telotarsus 0.20–0.21/0.03 (6.67–7.00).

#### Diagnosis

The new species belongs to the *hoffi* species-group and is characterised by the following combination of characteristics: trichobothria *b* and *sb* situated less than one areolar diameter apart; movable chelal finger with more than half of flattened teeth; chela 3.33–3.47 (♀), 3.56–3.73 (♂) and chelal hand 1.50–1.53 (♀), 1.56–1.67 (♂) longer than broad; chelal femur 0.38–0.39 mm (♀), 0.36–0.38 mm (♂), chelal hand 0.26–0.27 mm (♀), 0.24–0.25 mm (♂) and chelal movable finger 0.35–0.36 mm (♀), 0.33–0.35 mm (♂) long.

*Lechytiadepressidentata* **sp. nov.** differs from all other species of the *hoffi* species-group by trichobothria *b* and *sb* situated less than one areolar diameter apart.

#### Etymology

The specific name is derived from a combination of the Latin words “*depressus*” and “*dentatus*”, meaning flat and toothed, respectively, which refers to the teeth of movable finger including flat teeth.

#### Distribution

China (Xizang Autonomous Region).

#### Ecology

**Figure 12.**
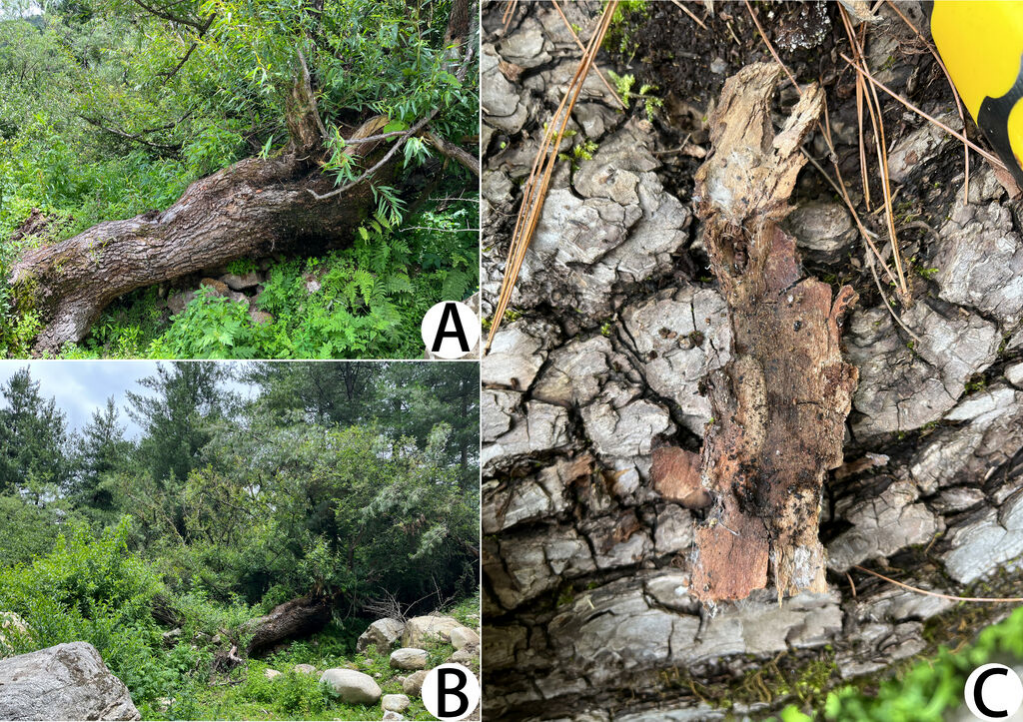
Type locality of *Lechytiadepressidentata* **sp. nov.**, **A–C** The habitat where specimens were collected.

The specimens of *Lechytiadepressidentata* **sp. nov.** were collected under bark in dense woods (Fig. 12).

#### Biology

No silk nest was found in the place where they were collected. Of course, this may be the result of inadequate sampling.

### Lechytia yulongensis

Zhang & Zhang, 2014

873A3EBD-2B49-57E3-963A-51F3DDEB571E

#### Materials

- **Type status:** Holotype. **Occurrence:** recordedBy: Aki Nakamura; sex: male; lifeStage: adult; occurrenceID: 66480400-CA62-583D-9866-7EE1B3F3D068; **Taxon:** scientificName: *Lechytiayulongensis*; nameAccordingTo: Zhang, F.B and Zhang, F. 2014. First report of the family Lechytiidae (Arachnida: Pseudoscorpiones) from China, with the description of a new species. Acta Zoologica Academiae Scientiarum Hungaricae, 60(3):217–225.; **Location:** country: China; stateProvince: Yunnan Province; locality: Jade Dragon Snow Mountains, Maoniuping; verbatimElevation: 3254 m; verbatimCoordinates: 27°08'N, 100°13'E; **Event:** year: 2012; month: August; day: 3; **Record Level:** institutionID: the Museum of Hebei University; institutionCode: Ps.-MHBU-YN12080301
- **Type status:** Paratype. **Occurrence:** recordedBy: Aki Nakamura; sex: male; lifeStage: adult; occurrenceID: 219D7E73-EF8A-5C45-BC3A-5BAABB6B44A6; **Taxon:** scientificName: *Lechytiayulongensis*; nameAccordingTo: Zhang, F.B and Zhang, F. 2014. First report of the family Lechytiidae (Arachnida: Pseudoscorpiones) from China, with the description of a new species. Acta Zoologica Academiae Scientiarum Hungaricae, 60(3):217–225.; **Location:** country: China; stateProvince: Yunnan Province; locality: Jade Dragon Snow Mountains, Maoniuping; verbatimElevation: 3254 m; verbatimCoordinates: 27°08′N, 100°13′E; **Event:** year: 2012; month: August; day: 3; **Record Level:** institutionID: the Museum of Hebei University; institutionCode: Ps.-MHBU-YN12080302
- **Type status:** Other material. **Occurrence:** recordedBy: Aki Nakamura; sex: female; lifeStage: adult; occurrenceID: 3A5C40DA-C533-5F2F-A918-61DE00CFB309; **Taxon:** scientificName: *Lechytiayulongensis*; nameAccordingTo: Zhang, F.B and Zhang, F. 2014. First report of the family Lechytiidae (Arachnida: Pseudoscorpiones) from China, with the description of a new species. Acta Zoologica Academiae Scientiarum Hungaricae, 60(3):217–225.; **Location:** country: China; stateProvince: Yunnan Province; locality: Jade Dragon Snow Mountains; verbatimElevation: 3240 m; verbatimCoordinates: 27.139°N, 100.229°E; **Identification:** identifiedBy: Jianzhou Sun; **Event:** year: 2012; month: August; habitat: conifer forest; **Record Level:** institutionID: the Museum of Hebei University; institutionCode: Ps.-MHBU-YN12080304

#### Description

**Figure 13.**
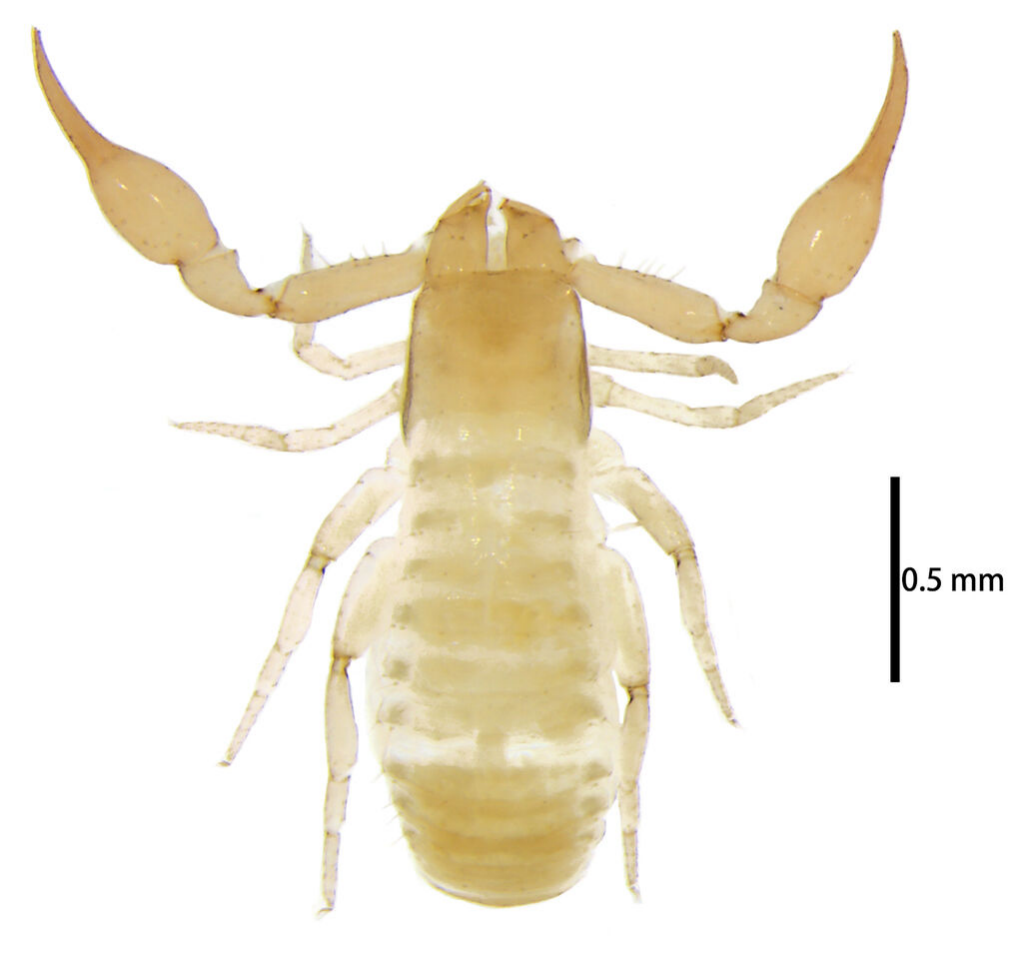
*Lechytiayulongensis*. Female (dorsal view). Scale bars: 0.50 mm.

**Figure 14.**
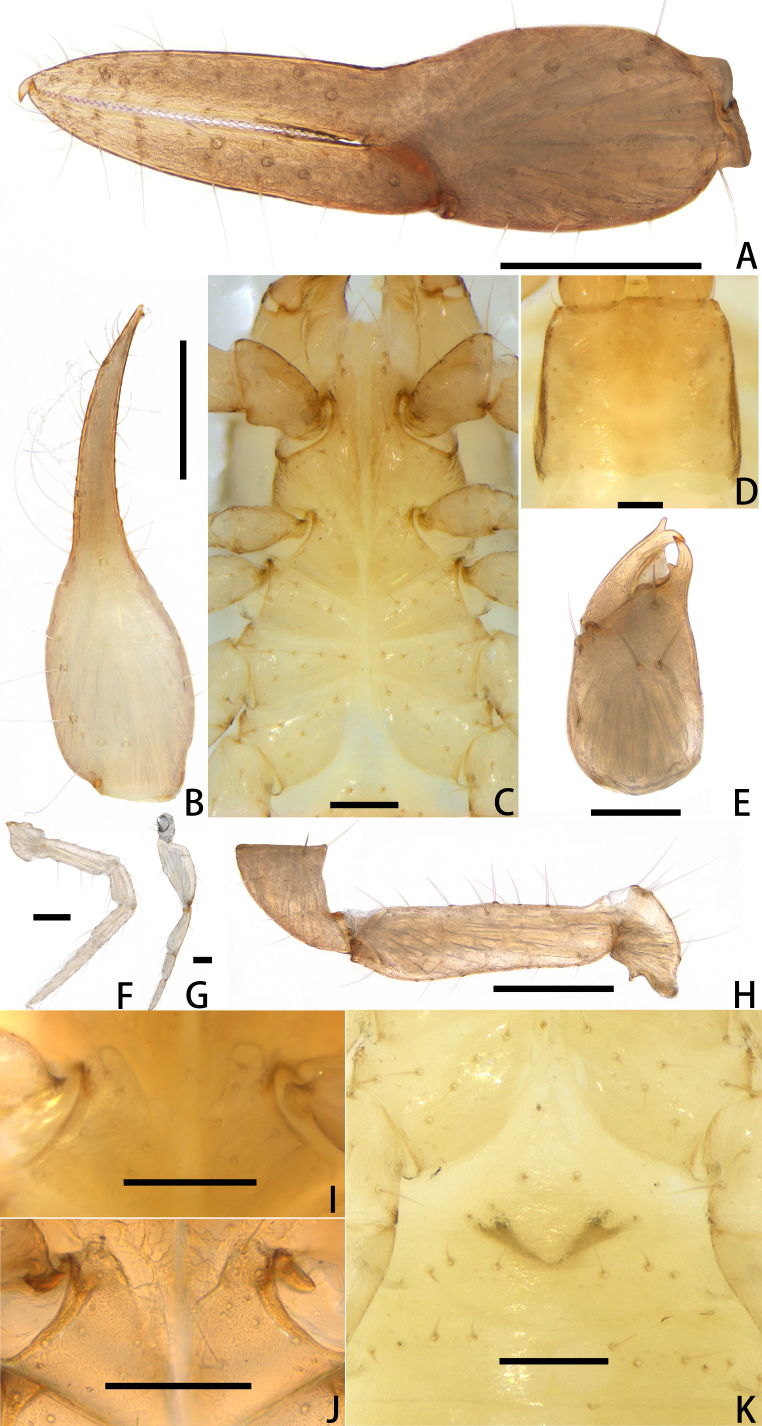
*Lechytiayulongensis*, female (A–I, K), holotype male (J): **A** Left chela (lateral view); **B** Left chela (dorsal view); **C** Coxal area and manducatory process (ventral view); **D** Carapace (dorsal view); **E** Left chelicera (dorsal view); **F** Leg Ⅰ (lateral view); **G** Leg Ⅳ (lateral view); **H** Left pedipalp (minus chela, dorsal view); **I** Apex of coxa I (female); **J** Apex of coxa I (male); **K** Female genital area (ventral view). Scale bars: 0.20 mm (A, B, H); 0.10 mm (C–G, I–K).

**Figure 15.**
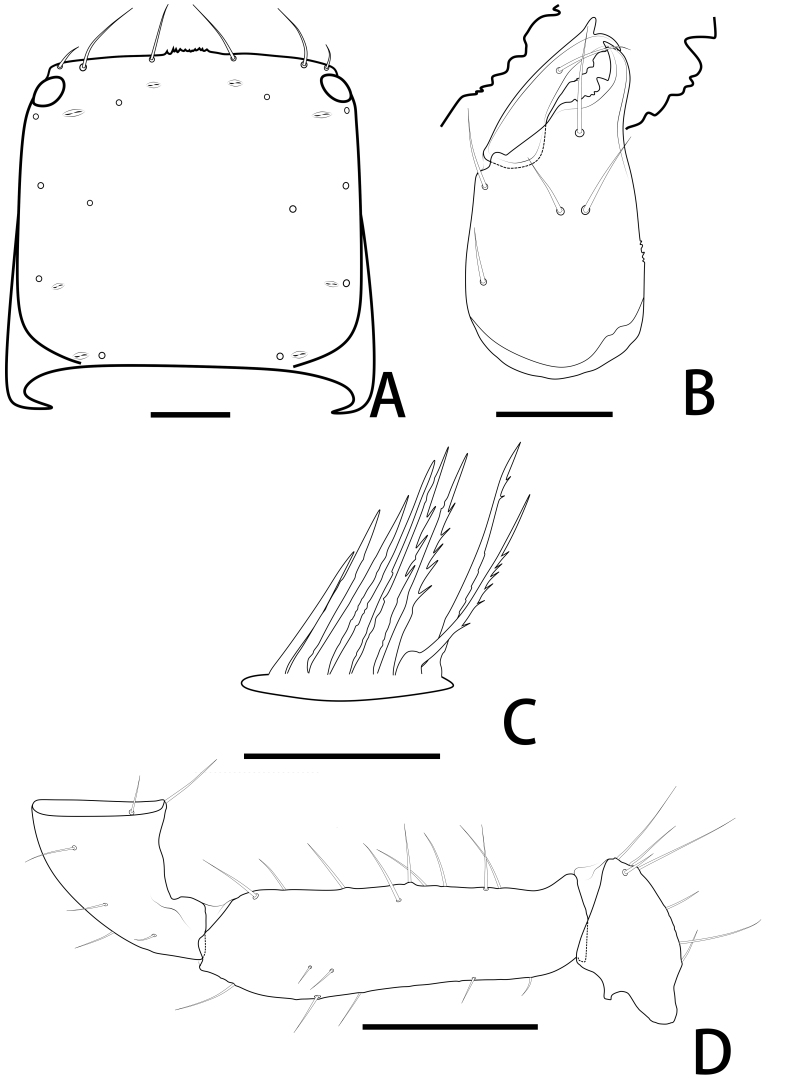
*Lechytiayulongensis*, female: **A** Carapace (dorsal view); **B** Left chelicera (dorsal view), with details of teeth; **C** Rallum; **D** Left pedipalp (minus chela, dorsal view). Scale bars: 0.10 mm (A–B); 0.05 mm (C); 0.20 mm (D).

**Figure 16.**
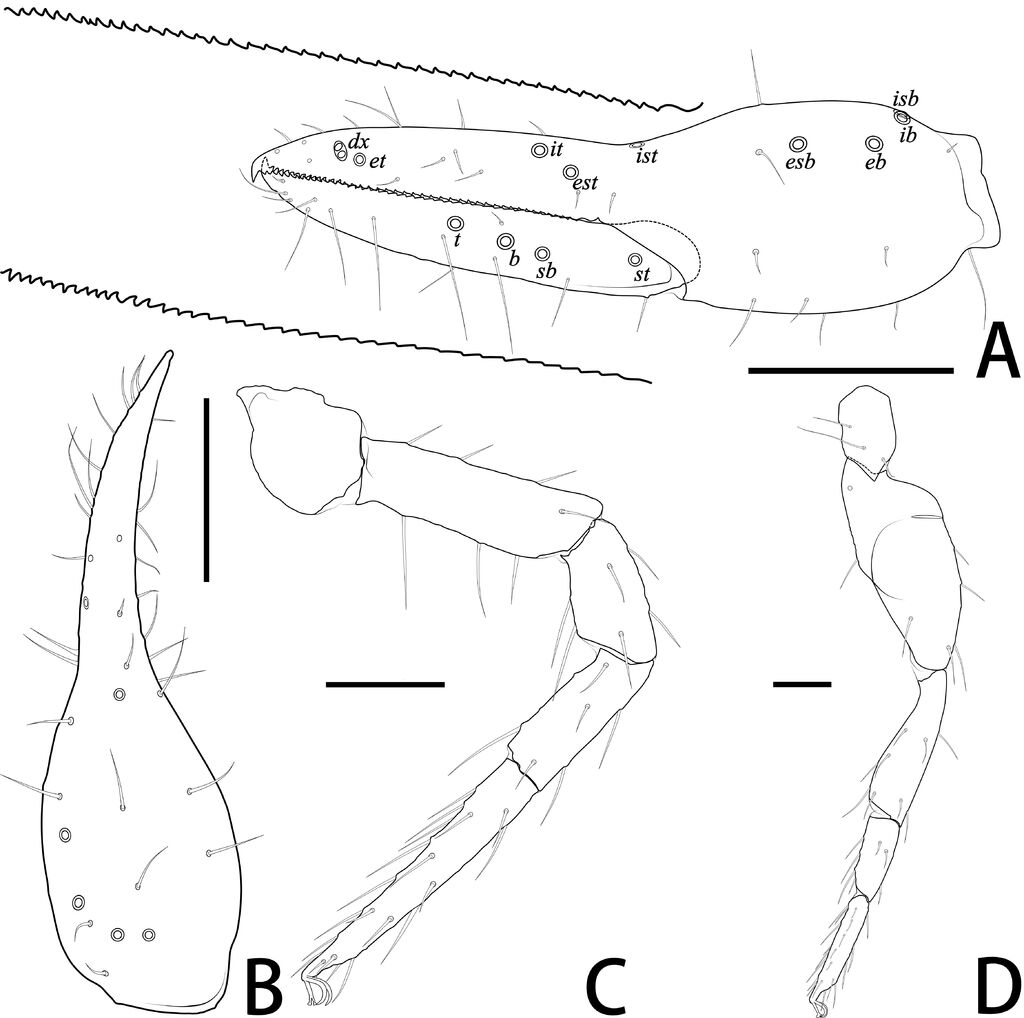
*Lechytiayulongensis*, female: **A** Left chela (lateral view), with details of teeth and trichobothrial pattern; **B** Left chela (dorsal view); **C** Leg I (lateral view); **D** Leg IV (lateral view). Scale bars: 0.20 mm (A, B); 0.10 mm (C, D).

Female (Fig. 13A, Fig. 14A–I, K and Figs 15, 16).

**Cephalothorax** (Fig. 14C, D, I and Fig. 15A): carapace nearly subquadrate, 0.87 times longer than broad; anterior margin denticulate; without epistome; two small corneate eyes; with 18 setae arranged 6: 4: 4: 2: 2, most setae heavy, long and gently curved; with four pairs of lyrifissures, first pair situated antero-medially, the second pair situated interno-underneath to the eyes, the third pair situated slightly interior to the sole pair of setae of the intermediate row and the fourth pair situated exterior to the sole pair of setae of the posterior row. Manducatory process with two acuminate distal setae, about equal in length, the distal setae terminally acuminate; apex of coxa I with a triangular apical projection; coxal spines and intercoxal tubercle absent. Chaetotaxy of coxae: P 5, Ⅰ 6, II 6, III 7, IV 6.

**Chelicera** (Fig. 14E and Fig. 15B): 1.80 times longer than broad; five setae present on hand, all setae acuminate, ventrobasal setae shorter than others; movable finger with one medial seta. Cheliceral palm has moderate wrinkles on both ventral and dorsal sides. Fixed finger with one large tooth and two roughened ridges proximally; movable finger with an acute apical tooth and four pointed, conspicuous middle teeth; galea shaped like a tooth (♂♀). Serrula exterior with 17 blades. Rallum with eight blades, subdistal blade strongly recumbent, others in straight row (Fig. 15C).

**Pedipalp** (Fig. 14A, B, H, Fig. 15D, Fig. 16A and B): trochanter 1.64, femur 3.31, patella 1.60, chela 3.45, hand 1.45 times longer than broad; femur 1.79 times longer than patella; movable chelal finger 1.48 times longer than hand and 0.62 times longer than chela. Setae generally long and acuminate. Fixed chelal finger and hand with eight trichobothria, *ib*, *isb*, *eb* and *esb* on dorsum of hand, *ib* and *isb* basally, *esb* submedially, *eb* closer to *ib* and *isb* than to *esb*; *ist* situated basally on fixed finger, *est* and *it* situated sub-basally and submedially on fixed finger, *et* and *dx* distally; movable chelal finger with four trichobothria, *b* closer to *sb* than to *t*, *sb* closer *b* than to *st*; *b* and *sb* situated more than one areolar diameter apart (Fig. 14A and Fig. 16A); sensilla absent. Both chelal fingers with a row of teeth: fixed finger with 41 developed retrorse, pointed teeth; movable finger with 37 developed retrorse, pointed teeth.

**Opisthosoma**: tergites and sternites undivided; setae acuminate. Tergal chaetotaxy I–Ⅻ: 6: 6: 6: 6: 6: 6: 6: 6: 6: 4: T2T: 0. Sternal chaetotaxy Ⅱ–Ⅻ: 6: 12: 12: 12: 10: 10: 9: 9: 10: -: 2. Genital area weakly sclerotised with U-shaped frame (Fig. 14K).

**Legs** (Fig. 14F, G, Fig. 16C and D): leg Ⅰ: femur 1.75 times longer than patella; tarsus 1.92 times longer than tibia. Leg Ⅳ: femoropatella 2.79 times longer than deep; tibia 3.00 times longer than deep; with sub-basal tactile setae on basitarsal segments. Arolium slightly shorter than the claws, not divided; claws simple.

**Dimensions** (length/breadth or, in the case of the legs, length/depth in mm; ratios in parentheses). **Female**: body length 1.59. Pedipalps: trochanter 0.18/0.11 (1.64), femur 0.43/0.13 (3.31), patella 0.24/0.15 (1.60), chela 0.69/0.20 (3.45), hand 0.29/0.20 (1.45), movable chelal finger length 0.43. Chelicera 0.27/0.15 (1.80), movable finger length 0.14. Carapace 0.39/0.45 (0.87). Leg I: trochanter 0.11/0.09 (1.22), femur 0.21/0.06 (3.50), patella 0.12/0.06 (2.00), tibia 0.13/0.05 (2.60), tarsus 0.25/0.04 (6.25). Leg Ⅳ: trochanter 0.16/0.12 (1.33), femoropatella 0.39/0.14 (2.79), tibia 0.24/0.08 (3.00), basitarsus 0.14/0.05 (2.80), telotarsus 0.25/0.03 (8.33).

#### Diagnosis

**Revised diagnosis** (♂♀). The species belongs to the *hoffi* species-group and is characterised by the following combination of characteristics: trichobothria *b* and *sb* situated more than one areolar diameter apart; movable chelal finger with strongly retrorse, pointed teeth; chela 3.45 (♀), 3.55–3.59 (♂) and chelal hand 1.45 (♀), 1.56–1.59 (♂) longer than broad; chelal femur 0.43 mm (♀), 0.40 mm (♂), chelal hand 0.29 mm (♀), 0.27–0.28 mm (♂) and chelal movable finger 0.43 mm (♀), 0.37–0.38 mm (♂) long.

*Lechytiayulongensis* closest to *L.acutidentata* **sp. nov.** due to trichobothria *b* and *sb* on movable chelal finger situated more than one areolar diameter apart. However, the species differs from *L.acutidentata* **sp. nov.** in the pattern of teeth of fixed chelal finger with retrorse and point teeth in *L.yulongensis*, but upright and point teeth in *L.acutidentata* sp. nov (Zhang and Zhang 2014).

#### Distribution

China (Yunnan Province).

#### Taxon discussion

*Lechytiayulongensis* was described and illustrated only from male specimens by Zhang and Zhang (2014). Although the original diagnosis contains the following character: trichobothria *b* and *sb* are only about one areolar diameter apart (Zhang and Zhang 2014), we found that the distance between trichobothria *b* and *sb* is more than one areolar diameter after inspection of the holotype, as shown in the original photo and illustration (Zhang and Zhang 2014: figs. 2C and 4A). In addition, we observed a triangular projection with a single hump on the apex of coxa I in the holotype of *L.yulongensis* (Fig. 14J), rather than a two-humped projection as shown in the original illustration (Zhang and Zhang 2014: 3A).

The female specimen was collected at the same place as the holotype, they have the same morphological characters, for example, the shape and number of chelal teeth and the positions of trichobothria. Therefore, we described the new specimen as female *L.yulongensis*. Based on the holotype and the new female specimen, we refined the diagnosis of *L.yulongensis* (see revised diagnosis) (Zhang and Zhang 2014).

## Identification Keys

### Key to the *hoffi* species-group of *Lechytia*

**Table:** 

1	Trichobothria *b* and *sb* situated more than one areolar diameter apart	2
–	Trichobothria *b* and *sb* situated almost or less than one areolar diameter apart	3
2	Movable chelal finger with upright and point teeth (Fig. 2A and Fig. 4A); pedipalpal femur length 0.49, chela 0.75, movable chelal finger 0.44.	*L.acutidentata* sp. nov.
–	Movable chelal finger with retrorse and point teeth (Fig. 14A and Fig. 16A) Zhang and Zhang (2014); pedipalpal femur 0.40, chela length 0.61–0.64, movable chelal finger 0.37–0.38.	*L.yulongensis* Zhang and Zhang 2014
3	Trichobothria *b* and *sb* situated less than one areolar diameter apart; movable chelal finger with more than half of flattened teeth (Fig. 8A and Fig. 10A).	*L.depressidentata* sp. nov.
–	Trichobothria *b* and *sb* situated almost 1 areolar diameter apart; movable chelal finger with small, triangular teeth at distal end, followed proximally by long, low teeth, nearly all with cusps (Muchmore 1975, figs. 16–17).	*L.hoffi* Muchmore 1975

## Analysis

These two new species, *L.acutidentata* and *L.depressidentata*, can be placed in the *hoffi* species-group, based on the following characteristics: well-developed chelal teeth, simple distal seta on pedipalpal coxa, tergite XI with chaetotaxy T2T,and male galea nearly as well developed as in female. The four species of *hoffi* species-group can be distinguished by the following characteristics: the distance between trichobothria *sb* and *b* on movable chelal finger, the tooth pattern of movable chelal finger and the length of pedipalpal podomeres.

## Supplementary Material

XML Treatment for Lechytia acutidentata

XML Treatment for Lechytia depressidentata

XML Treatment for Lechytia yulongensis
